# Formulation and candidacidal activity of magnetic nanoparticles coated with cathelicidin LL-37 and ceragenin CSA-13

**DOI:** 10.1038/s41598-017-04653-1

**Published:** 2017-07-04

**Authors:** Katarzyna Niemirowicz, Bonita Durnaś, Grażyna Tokajuk, Ewelina Piktel, Grzegorz Michalak, Xiaobo Gu, Alina Kułakowska, Paul B. Savage, Robert Bucki

**Affiliations:** 1 Department of Microbiological and Nanobiomedical Engineering, Medical University of Bialystok, 15-222 Białystok, Poland; 2 Department of Microbiology and Immunology, The Faculty of Health Sciences of the Jan Kochanowski University in Kielce, 25-317 Kielce, Poland; 3 Department of Periodontal and Oral Mucosa Diseases, Medical University of Bialystok, 15-269 Białystok, Poland; 4 Department of Chemistry and Biochemistry, Brigham Young University, Provo, UT USA; 5 Department of Neurology, Medical University of Białystok, 15-276 Białystok, Poland

## Abstract

Fungal infections caused by *Candida* spp. represent an emerging problem during treatment of immunocompromised patients and those hospitalized with serious principal diseases. The ever-growing number of fungal strains exhibiting drug resistance necessitates the development of novel antimicrobial therapies including those based on membrane-permeabilizing agents and nanomaterials as drug carriers. In this study, the fungicidal activities of LL-37 peptide, ceragenin CSA-13 and its magnetic derivatives (MNP@LL-37, MNP@CSA-13) against laboratory and clinical strains of *C. albicans, C. glabrata* and *C. tropicalis* were evaluated. These experiments confirm the high anti-fungal activity of these well-characterized agents mediated by their interaction with the fungal membrane and demonstrate elevated activity following immobilization of LL-37 and CSA-13 on the surface of magnetic nanoparticles (MNPs). Furthermore, MNP-based nanosystems are resistant to inhibitory factors present in body fluids and effectively inhibit formation of fungal biofilm. Simultaneously, synthesized nanostructures maintain immunomodulatory properties, described previously for free LL-37 peptide and CSA-13 substrate and they do not interfere with the proliferation and viability of osteoblasts, confirming their high biocompatibility.

## Introduction

Over the last 40 years the number of fungal infections has continued to grow. More than 70% of these cases are caused by *Candida* spp, especially in the large population of patients with immune disorders and/or those hospitalized with serious underlying diseases^1, 2^. *Candida* are the most common fungal pathogens and cause diseases ranging from superficial (oral and vaginal) to systemic (peritonitis, meningitis fungemia) candidiasis^3, 4^. The yeast genus is composed of a heterogeneous group of organisms, and more than 17 different *Candida* species are reported as etiological agents of human infection. However, among the different species of *Candida* more than 90% of invasive infections are caused by *Candida albicans*, *Candida glabrata* and *Candida tropicalis* ^5^. Biofilm formation plays an important role in the development of yeast infections. Moreover, the ability of *Candida* species to form biofilms is an important aspect of developing drug-resistance^6^. In over 60% of cases, chronic or recurrent candidiasis can develop in immunocompromised hematological patients as a consequence of chronic antibiotic therapy^7–10^. Bacterial coexistence represents an important problem during fungal infection, which enhances the inflammatory response and complicates treatment. Concomitant bacteremia is associated with a poor prognosis despite antimicrobial therapy compared with monomicrobial candidemia^11^. Therefore, the treatment of yeast infections often requires the use of combined therapy, including additional therapeutic substances from the group of antibiotics and steroids. This therapy often results in the accumulation of adverse side effects. Despite a high number and diverse group of currently available antibacterial drugs, the number of active substance for treatment of pathogenic fungi is quite low. Recent reports show that a growing number of *Candida*-mediated infections are resistant to the current classes of antifungal agents particularly the azoles (where resistance has climbed most prominently). Therefore, the search for new treatments for yeast infections is an important challenge for modern medicine.

The rapid development of nanotechnology is expected to have a dramatic impact on medicine^12^. Recent data indicate that nontraditional antimicrobial agents have great promise in the treatment of infectious disease. Specifically, certain classes of nanomaterials, initially proposed as drug carriers in the delivery of chemotherapeutic agents, have been investigated in both *in vitro* and *in vivo* settings^13–16^. Metallic nanoparticles such as silver, gold, selenium, and ferrum are characterized by inherent antimicrobial properties^17^. The bactericidal properties of different nanostructures include: pure metal (Au, Ag, Fe), alloys (CdFe, FePt), oxides (Fe_3_O_4_, SeO) as well as core-shell structures are reported as effective agents that restrict the growth of pathogenic bacteria^18^. The application of nanotechnology to provide new methods of fungal infection treatment include silver, titanium dioxide and zinc oxide nanoparticles^19, 20^. However, there are limited studies indicating the effect of iron-oxide magnetic nanoparticles (MNPs) either alone or as drug carriers against fungal pathogens such as *Candida* ^13, 21^. In the present study we evaluate the activity of magnetic nanosystems functionalized with LL-37 peptide or cationic lipids against clinical isolates of *Candida* species. The hypothesis guiding this study assumes that the unique properties of magnetic nanoparticles (MNPs) functionalized with antifungal antibiotics enables the optimization of the fungicidal effect. We observe that immobilization of cationic lipids on the magnetic carrier enhances their antifungal activity compared to unattached compounds in the presence of different body fluids. Furthermore, these nanosystems are characterized by lower lytic activity against osteoblasts indicating their improved biocompatibility. In addition, nanoparticles functionalized by cationic lipids did not increase the release of the proinflammatory cytokine IL-8 and do not interfere with cell proliferation at concentration sufficient to induce antifungal effect.

## Results

### Nanoparticle characterization

**Figure 1:**
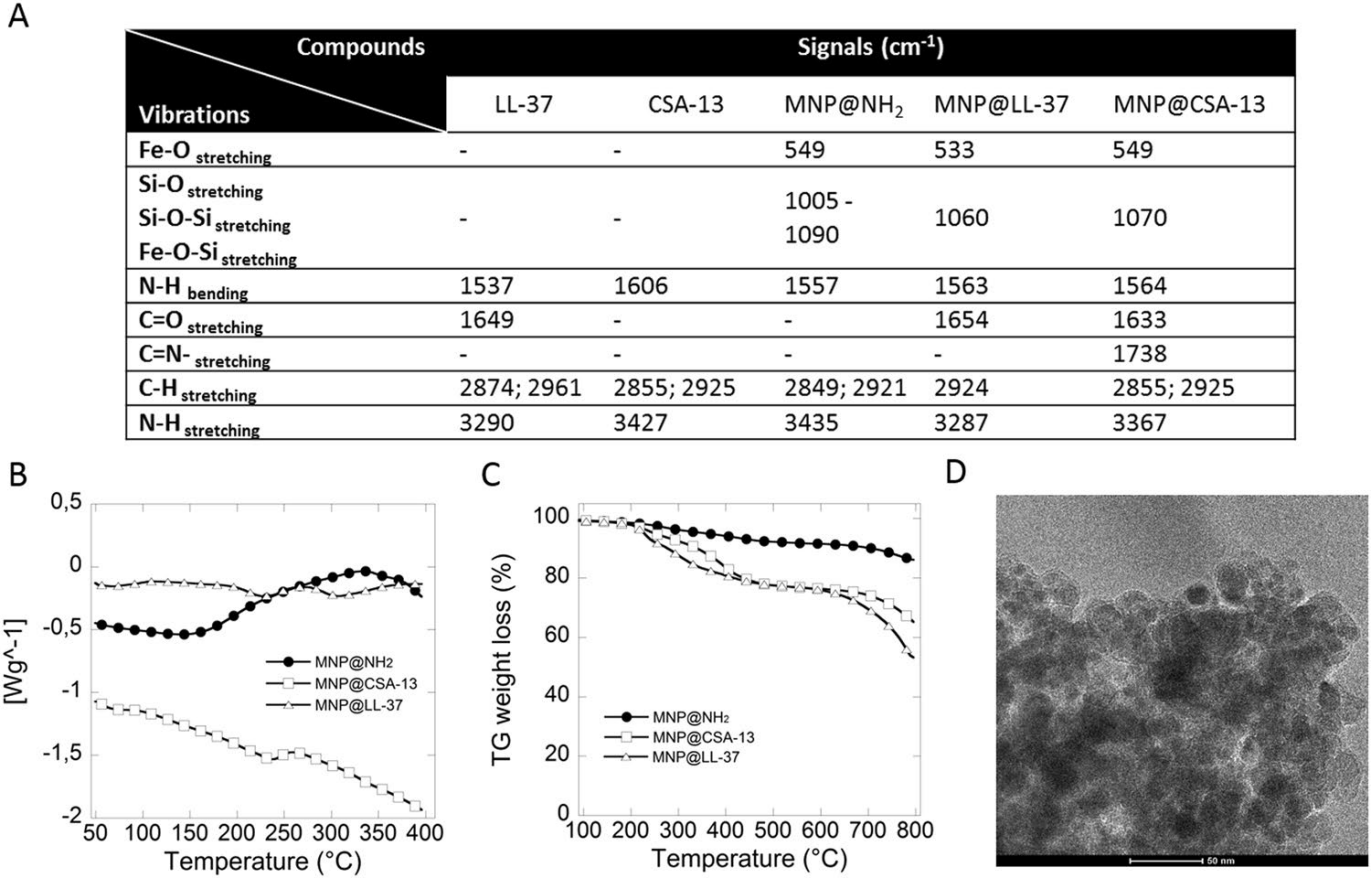
Physicochemical features of functionalized magnetic nanoparticles. Panel A summarized vibrations measured for unfunctionalized magnetic nanoparticles (MNP@NH_2_), and antimicrobials (LL-37 and CSA-13) in free and immobilized form (MNP@LL-37 and MNP@CSA-13). Heating curves of MNP@NH_2_ (filed circles), MNP@LL-37 (white triangles) and MNP@CSA-13 (white squares) shows in panel B. Thermogravimetric weight loss of MNP@NH_2_ (filed circles), MNP@LL-37 (white triangles) and MNP@CSA-13 (white squares) shows in panel C. Representative TEM images of cationic lipids functionalized magnetic nanoparticles (panel D).

The ATR FT-IR spectra show several characteristic functional group bands including: siloxal bands, imine bond, imide in-plane bands/carbonyl bond. C-H stretching modes further confirm the presence of a silica shell on the MNP surface and CSA-13/LL-37 immobilization. The characteristic bands for aminosilane coated magnetic nanoparticles, CSA-13 and LL-37 in free and immobilized form are summarized in Fig. 1 panel A. Calorimetric analysis of heating curves shows changes in their course indicating differences in the chemical nature of tested agents, which confirms successful immobilization of CAPs on the MNP surface (Fig. 1B). Thermogravimetric analysis (TGA) of CAP-functionalized MNPs demonstrates a total weight loss of 35% and 45% for CSA-13 and LL-37, respectively. Figure 1C shows that the weight loss of the CAP-decorated nanosystem compared to the weight loss of bare and aminosilane nanoparticles indicates effective CAPs immobilization on the MNP surface, with approximately 15–25% CAP content. Both particles are spherical with a diameter of 15 nm ± 2 nm. These values were calculated using 100 randomly selected particles from TEM images (Fig. 1D) and confirmed by XRD technique (data not shown)^22^. Based on TGA-weight lost and total amount of amine groups on the MNP surface, we calculated that the number of CSA-13 molecules was 3.53 × 10^13^, which corresponds to a number of molecules 20 times lower per 1 µg of compound^22, 23^. However in the case of LL-37, we calculated that the number of cathelicidin molecules was 6.32 × 10^13^, which corresponds to a number of molecules 2.2 times lower per 1 µg of LL-37.

### Nanoparticles decrease the MIC/MFC/MBIC value of cationic lipids derivatives and enhance their fungicidal activity in the presence of body fluids

MIC/MFC/MBIC values are initially used to determine the therapeutic potential of novel antimicrobial agents. In order to assess these values, 18 laboratory and clinical isolates of *Candida* spp. were used. The data presented in Table 1 confirm that ceragenin CSA-13 possesses high candidacidal activity with MIC values ranging from 0.5 to 4 µg/mL. In contrast, the fungicidal activity of LL-37 and bare MNPs is significantly lower (32 to > 256 µg/mL). Importantly, immobilization of LL-37 and CSA-13 on the surface of magnetic carriers (MNP@LL-37 and MNP@CSA-13) significantly increases the killing properties of these compounds against all tested fungal strains. The largest decrease in MIC/MFC/MBIC values was observed for LL-37 peptide; the use of MNP@LL-37 rather than immobilized LL-37 increased the biological activity of this compound against laboratory and clinical *Candida* strains from 2 to 32-fold.

**Table 1:** Minimal inhibitory concentration (MIC, µg/ml), minimal fungicidal concentration (MFC, µg/ml) and minimal biofilm inhibitory concentration (MBIC) of CSA-13, LL-37, MNPs and their derivatives (MNP@CSA-13 and MNP@LL-37) against laboratory and clinical isolates (*) of *Candida* spp.

Strain	MIC/MFC/MBIC [µg/ml]				
	CSA-13	LL-37	MNP	MNP@CSA-13	MNP@LL-37
*C. albicans*	1/1/4	>256/>256/>256	64/128/256	0.5/0.5/2	8/16/32
*C. albicans*	4/4/8	>256/>256/>256	64/64/256	4/4/8	32/32/128
*C. albicans*	0.5/1/4	64/128/256	128/256/>256	0.5/1/2	32/64/64
C. albicans*	2/2/8	128/256/>256	64/128/256	0.5/1/2	16/32/64
C. albicans*	4/8/16	256/>256/>256	64/64/128	0.5/1/4	32/32/64
C. albicans*	2/2/8	>256/>256/>256	>256/>256/>256	0.5/1/2	32/64/128
C. albicans*	0.5/1/2	128/256/>256	128/128/>256	0.5/1/2	64/128/256
C. albicans*	2/4/16	256/>256/>256	128/256/>256	1/2/4	32/64/128
C. albicans*	1/2/4	256/>256/>256	128/128/256	1/2/2	32/64/128
C. albicans*	4/8/16	256/>256/>256	64/128/>256	1/2/4	16/32/64
C. glabrata*	2/2/8	>256/>256/>256	64/128/256	2/2/4	64/128/256
C. glabrata*	1/2/4	32/64/128	256/>256/>256	0.5/1/2	4/8/32
C. glabrata*	0.5/1/2	>256/>256/>256	256/>256/>256	0.5/0.5/1	32/64/128
C. tropicalis*	0.5/1/2	>256/>256/>256	256/>256/>256	0.5/1/1	16/32/128
C. tropicalis*	1/2/4	>256/>256/>256	64/128/256	0.5/1/2	16/32/64
C. tropicalis*	0.5/1/2	64/128/256	64/128/256	0.5/1/1	8/16/64
C. tropicalis*	0.5/1/2	64/256/>256	64/128/256	0.5/0.5/1	16/32/64
C. spp*	0.5/1/2	64/128/256	>256/>256/>256	0.5/0.5/1	16/16/64

Intensification of LL-37 and CSA-13 candidacidal activity following the formation of MNP-based derivatives is also observed in the presence of body fluids collected from 7 different compartments. As presented in Table 2, CSA-13 maintains its fungicidal properties in all body fluids; however, considerable variations in killing activity of this ceragenin were detected for samples incubated in the presence of urine, pus, and dental plaque. Importantly, MNP@CSA-13 is characterized by strong candidacidal activity against selected *Candida* spp. strains, despite the presence of body fluids comprising a number of factors that might inhibit its activity. A similar influence of MNPs was observed for human cathelicidin, however, this effect was considerably reduced when compared to a synthetic analog of LL-37 peptide.

**Table 2:** Minimal inhibitory concentration (MIC, µg/ml), minimal fungicidal concentration (MFC µg/ml) and minimal biofilm inhibitory concentration (MBIC) of CSA-13, LL-37, MNPs and their derivatives (MNP@CSA-13, MNP@LL-37) against selected clinical isolates of *Candida* spp. in the presence of different body fluids.

Strain	CSA-13 (MIC/MFC/MBIC)	LL-37 (MIC/MFC/MBIC)	MNP (MIC/MFC/MBIC)	MNP@CSA-13 (MIC/MFC/MBIC)	MNP@LL-37 (MIC/MFC/MBIC)
***C. albicans***	4/8/16	256/>256/>256	64/128/>256	1/2/4	16/32/64
Saliva	2/4/8	128/256/>256	64/128/256	1/1/2	16/32/64
Urine	32/64/128	>256/>256/>256	256/>256/>256	4/4/8	32/64/128
Plasma	4/8/16	256/>256/>256	64/128/256	2/4/8	32/64/128
Pus	32/64/128	>256/>256/>256	256/>256/>256	4/8/16	32/64/128
Abdominal fluid	4/8/16	256/>256/>256	64/128/256	1/2/4	32/64/128
Cerebrospinal fluid	2/4/16	256/>256/>256	128/256/>256	1/1/4	32/64/128
Dental plaque	16/32/32	>256/>256/>256	128/256/256	8/8/16	64/128/128
***C. glabrata***	0.5/1/2	>256/>256/>256	256/>256/>256	0.5/0.5/1	32/64/128
Saliva	2/2/4	>256/>256/>256	>256/>256/>256	2/4/4	32/64/128
Urine	4/4/16	>256/>256/>256	>256/>256/>256	4/8/8	64/64/128
Plasma	2/2/4	>256/>256/>256	256/>256/>256	1/2/4	128/128/256
Pus	8/8/16	>256/>256/>256	>256/>256/>256	4/8/8	256/256/>256
Abdominal fluid	1/2/2	>256/>256/>256	256/>256/>256	0.5/1/1	128/128/256
Cerebrospinal fluid	1/1/2	>256/>256/>256	256/>256/>256	0.5/1/1	32/64/64
Dental plaque	8/8/16	>256/>256/>256	>256/>256/>256	4/4/8	128/256/>256
***C. tropicalis***	0.5/1/2	64/256/>256	64/128/256	0.5/0.5/1	16/32/64
Saliva	4/8/16	128/256/>256	128//256/>256	1/1/2	32/64/128
Urine	8/8/32	256/>256/>256	64/128/>256	4/8/16	32/64/128
Plasma	1/2/4	64/128/>256	64/128/256	0.5/1/2	16/32/64
Pus	8/16/32	256/>256/>256	256/>256/>256	4/4/8	128/256/256
Abdominal fluid	2/4/8	256/>256/>256	128/256/>256	1/1/2	16/32/32
Cerebrospinal fluid	1/1/2	64/128/256	128/256/256	0.5/1/2	16/32/64
Dental plaque	8/16/32	>256/>256/>256	256/>256/>256	4/4/8	64/64/128

### Cationic lipids (LL-37 and CSA-13), bare magnetic nanoparticles (MNPs) and their derivatives (MNP@CSA-13, MNP@LL-37) prevent cells adhesion and formation of fungal biofilm

**Figure 2:**
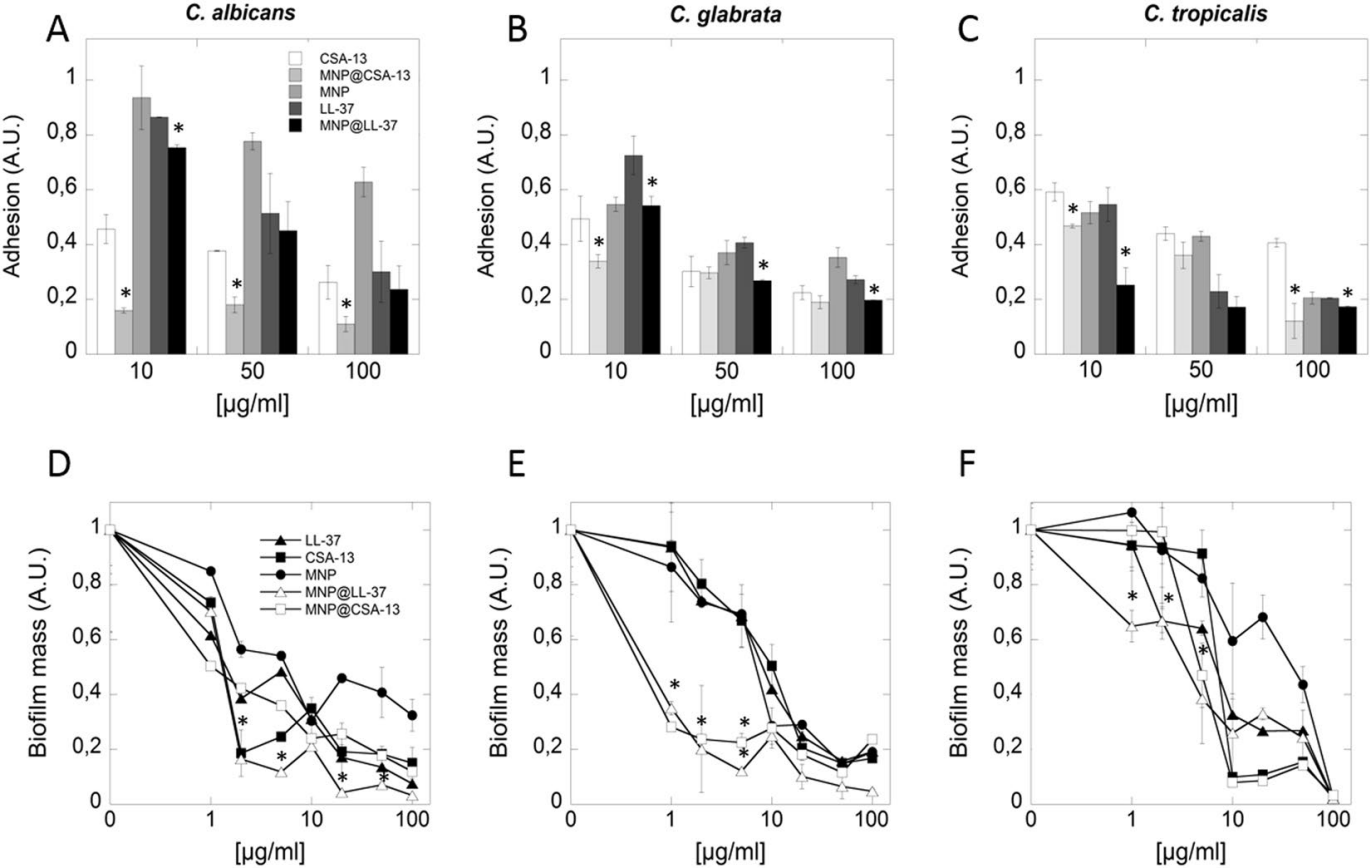
Cathelicidin LL-37, ceragenin CSA-13, bare magnetic nanoparticles (MNPs) and their derivatives (MNP@CSA-13, MNP@LL-37) prevent adhesion and formation of fungal biofilm. Inhibition of adhesion of clinical isolates of *Candida albicans* (panel A), *Candida glabrata* (panel B) and *Candida tropicalis* (panel C) during treatment with cationic lipids and their magnetic derivatives. Fungicidal activity of cationic lipids and their magnetic derivatives against biofilm forming clinical isolates of *Candida species* (panels D–F). Results represent mean ± SD from 3 experiments. *p < 0.05 – statistical significance to agents in free form.

Reduction of fungal adhesion and inhibition of biofilm growth are fundamental challenges for modern medicine. We therefore assessed the ability of the tested agents to modulate fungal adhesion and biofilm formation (Fig. 2). The adhesion of *Candida albicans* cells is reduced by 40% in the presence of MNP@CSA-13 compared to free CSA-13 (Fig. 2A). When cathelicidin is immobilized on MNPs a three times lower adhesion of *Candida tropicalis* cells is observed (Fig. 2C). Additionally, data in Fig. 2D–F indicates that CAPs attached to MNPs have an increased ability to restrict the growth of mature biofilms compared to non-immobilized agents. The two to four fold enhanced inhibition of biofilm formation was observed after addition CAPs magnetic derivatives at a concentration range of 1–10 μg/mL.

### Magnetic nanoparticles optimize the interaction of cationic lipids with fungal membranes and trigger oxidative damage of Candida cells

**Figure 3:**
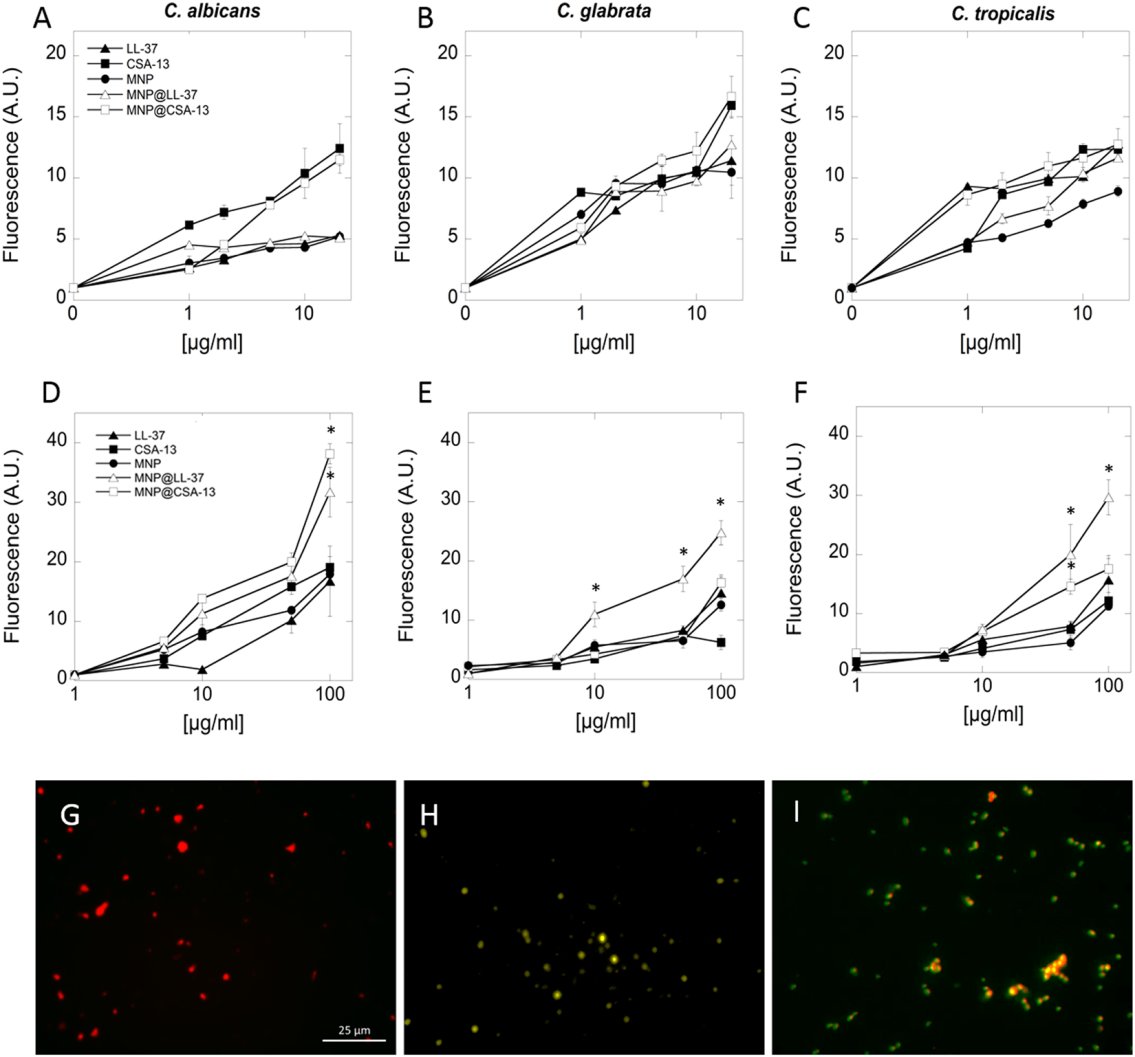
Interactions of cathelicidin LL-37, ceragenin CSA-13, bare magnetic nanoparticles (MNPs) and their derivatives (MNP@CSA-13, MNP@LL-37) with the fungal membrane. Disruption of the Candida cells membrane integrity (panels A–C). Kinetics of FITC-labeled cationic lipids and their magnetic derivatives insertion into the fungal cell plasma membrane (panels D–F). Representative confocal images indicated internalization of FITC-functionalized nanosystems into Candida cells (**G** – control cells; **H** – after addition 50 μg/mL MNP@LL-37; **I** - after addition 50 μg/mL MNP@CSA-13). Results represent mean ± SD, n = 3. *p < 0.05 – statistical significance to agents in free form.

**Figure 4:**
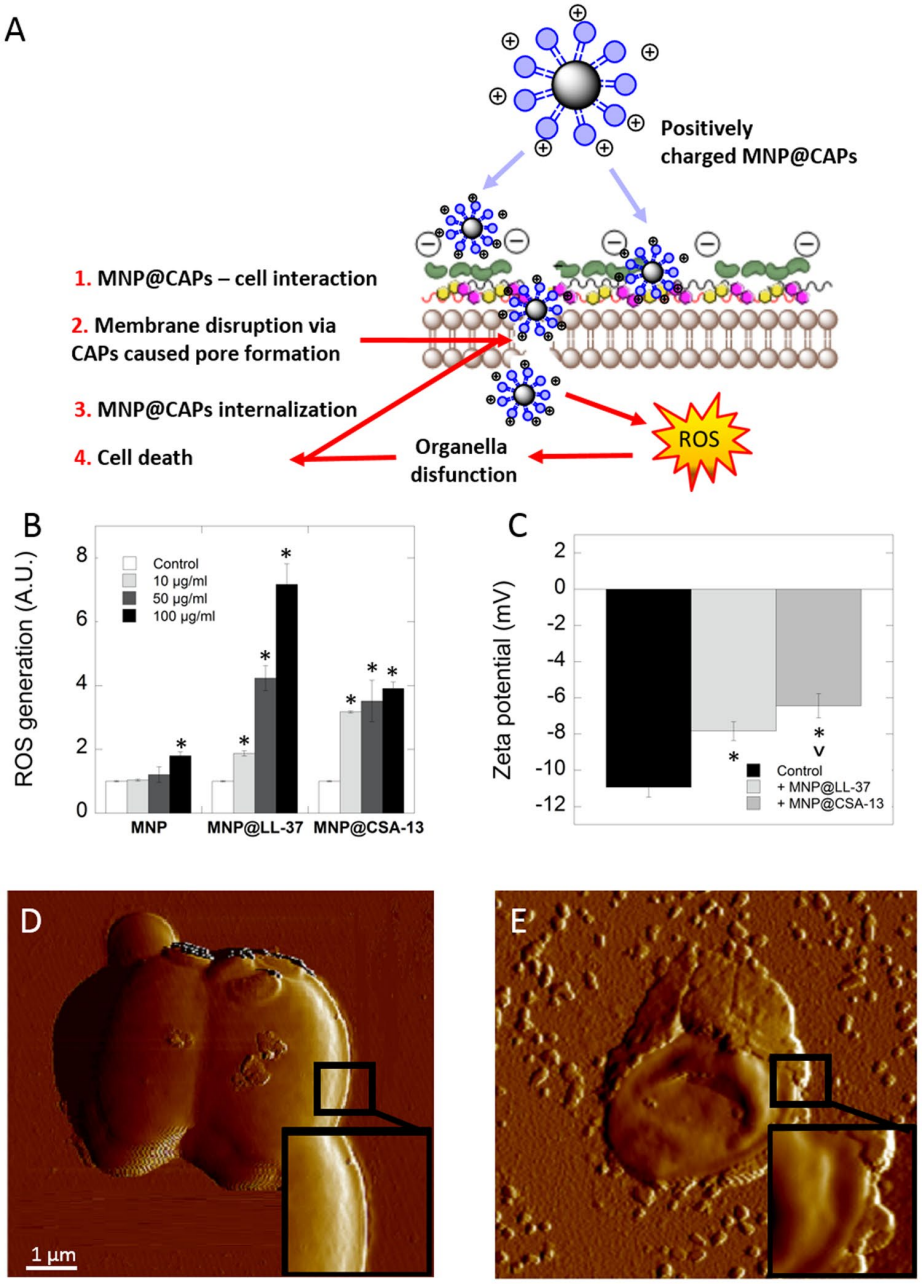
LL-37 and CSA-13 coated magnetic nanoparticles caused destruction of fungal cells membrane and contribute to ROS generation. Hypothetical mechanism of positively charged magnetic nanoparticles functionalized by cationic lipids against fungal cells (**A**). Magnetic nanosystems contribute to generation of ROS in *Candida albicans* cells (**B**). Changes in zeta potential of negatively charged fungal cells after addition of cathelicidin LL-37 and ceragenin CSA-13 decorated magnetic nanoparticles (**C**). Topography (AFM) of untreated (**D**) and treated (**E**) *Candida albicans* cells with MNP@CSA-13 (10 μg/mL). *p < 0.05 – statistical significance to control; ^p < 0.05 – statistical significance to MNP@LL-37.

A key feature of cationic lipids is their non-specific interaction with fungal membranes, a quality that maintains fungal membrane destruction and prevents the development of fungal resistance. Results shown in Fig. 3A–C demonstrate that the tested agents in both free as well as immobilized form were able to disrupt outer fungal membrane in dose-dependent manner. Furthermore, a dose-dependent increase in the uptake of MNP functionalized by cationic lipids compared to agents in free form was observed. A two-fold greater stronger interaction of magnetic derivatives with yeast cells compared to free compounds was observed (Fig. 3D–F). To confirm the ability of CAPs immobilized on magnetic nanoparticles to internalize into fungal cells, real-time confocal microscopy analysis was performed (Fig. 3G–I). For the purpose of detailed evaluation of fungicidal activity of tested nanosystems, the abilities of these systems to contribution to generation reactive oxygen species (ROS) were measured, zeta-potential was analyzed and fungal topography was recorded by atomic force microscopy. Figure 4B shown that treatment of representative clinical isolates of *Candida albicans* with magnetic nanoparticles contributes to generation of ROS. Interestingly, addition of aminosilane coated nanoparticles used as a drug carrier caused lower oxidative damage than coated nanoparticles (Fig. 4B). Data indicated that electrokinetic mobility of the *Candida albicans*, as measured through cell surface charge changes from -11 eV to -8 eV or to -6 eV after addition of MNP@LL-37 and MNP@CSA-13, respectively at concentration of 100 μg/mL (Fig. 4C). In turn, to show the effects of these nanosystems on *Candida albicans* cell morphology, atomic force microscopy (AFM) was performed. Figure 4D,E shows the representative topography of fungal cells before and after treatment with MNP@CSA-13. Compared to untreated controls, extensive morphological alternations in fungal cells treated with nanosystems containing cationic lipids were observed. The collected data indicate nanosystem attachment and penetration into *Candida* cells associated with disruption of the cell wall. As a consequence of this interaction, the wrinkled surface of fungal cell as well as a leakage of the intracellular contents was observed. Additionally a change of cell shape was observed. Based on these results we propose the mechanism of action of these nanosystems shown in Fig. 4A.

### Magnetic nanoparticles functionalized with CAPs display low toxicity to human osteoblast cells

**Figure 5:**
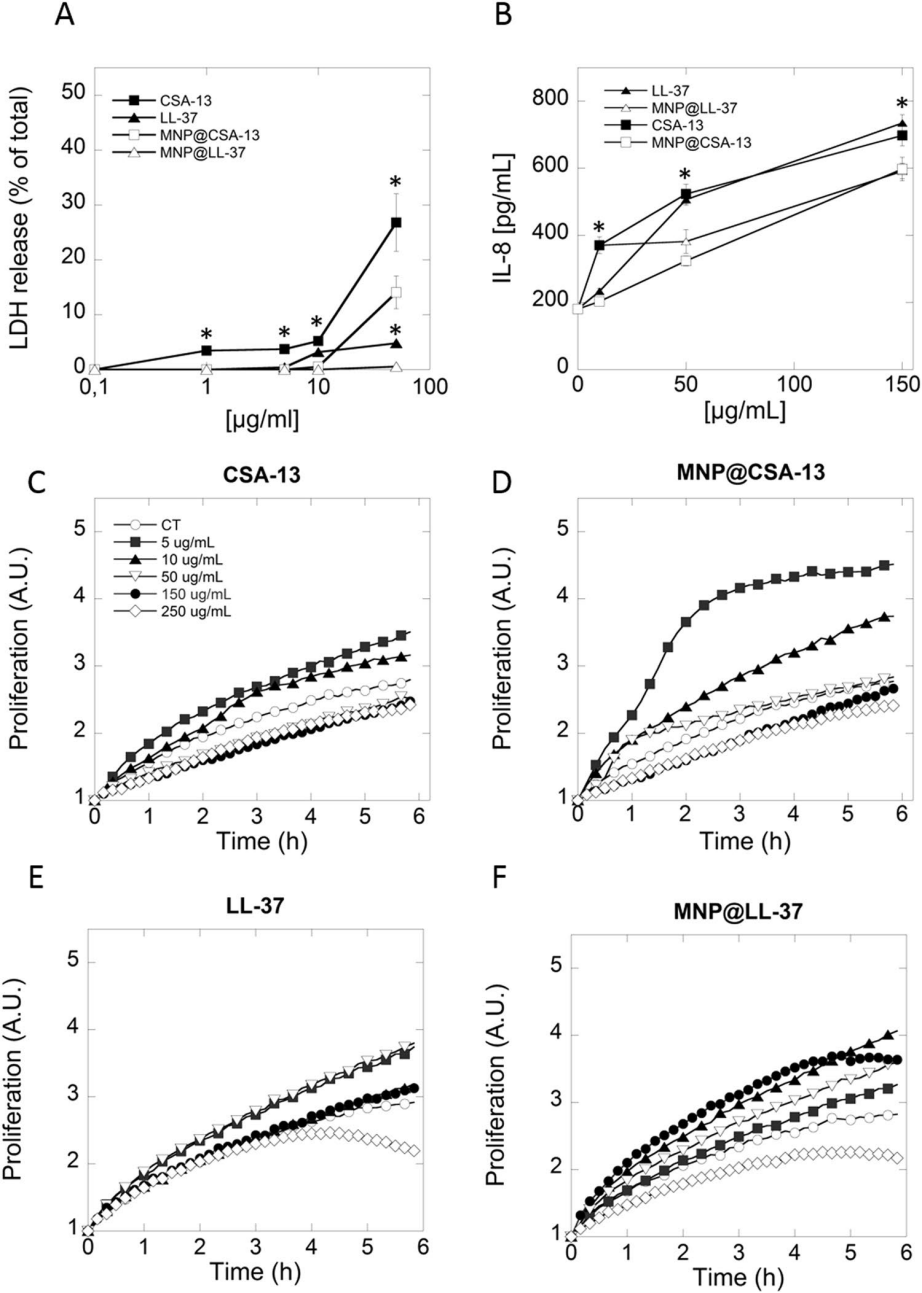
Effect of cathelicidin LL-37, ceragenin CSA-13, bare magnetic nanoparticles (MNPs) and their derivatives (MNP@CSA-13, MNP@LL-37) on human osteoblast cells integrity, IL-8 release, and their growth. Panel A and B show release of LDH and IL-8 from human osteoblast after 24 h of treatment and panels C–F show their growth. Results represent mean ± SD from 3 experiments. *p < 0.05– statistical significance to agents in immobilized form.

As shown in Fig. 5A, the tested MNP derivatives do not cause disruption of osteoblast membranes within a concentration range from 1–50 µg/mL. Compared to free CSA-13, which causes lysis of more than 25% of the osteoblast cells at 50 µg/mL, the toxicity of MNP@CSA-13 was significantly lower. The functionalization of MNPs with LL-37 also improves biocompatibility of this peptide, resulting in decreased LDH release to almost non-detectable levels. Figure 5B indicate an increase in IL-8 release from human osteoblast cells following non-immobilized CAPs addition at concentration ranges similar to those required for effective fungal killing. Among the tested agents, CSA-13 showed the strongest ability to inhibit hFOB 1.19 cell proliferation, causing a 20% decrease after 6 h of incubation at 50 µg/mL (Fig. 5C). A similar effect was observed after addition of LL-37 at 250 µg/mL. When MNP@CSA-13 and MNP@LL-37 were applied to hFOB 1.19 cells at the same concentration a decrease in proliferation of 5% and 12% were observed, respectively. These values are non-significant. Importantly, the tested nanosystems are able to enhance cell proliferation which provides evidence for their potential benefit in cell regeneration systems.

## Discussion

The growing number of resistant fungal strains demands the development of new therapeutic tools and novel treatment methods. To date, a number of studies have established the usefulness of nanotechnology-based approaches in development of antibacterial and antifungal substances^24, 25^. Among the nanomaterials evaluated for these purposes, nanostructures based on silver, titanium dioxide, zinc oxide and iron oxide demonstrate the greatest promise^19, 20, 26^. Interestingly, this phenomenon is only not limited to the nanomaterial-based conjugates, since bare, unfunctionalized nanoparticles also exhibit considerable antibacterial activity. To date, it is established that the mechanism of antimicrobial activity of magnetic nanoparticles involves generation of reactive oxygen species (ROS) followed by disruption in bacterial electron transport of oxidation of NADH. Additionally, it was reported that bacteria eradication might be achieved via damage of macromolecules including DNA, lipids and protein after MNP treatments^27^. Our studies focused on candidiasis treatment using MNPs-based nanoagents. We reported that incubation of fungal cells with MNPs inactivate the catalase Cta1 and disturb the oxidation-reduction balance^26^. The observation that MNPs coated with LL-37 or CSA-13 increased generation of ROS is probably associated with mechanism of action of CAPs that results in pore formation within cell membranes, also contributing to nanoparticle transport into *Candida* cells. In effect, destruction of fungal cell wall integrity enhanced MNP internalization and exertion of oxidative damage. Moreover, it is hypothesized that both CAPs and magnetic nanoparticles affect the redox status of cells, resulting in potentiation of ROS formation. Liu *et al*. suggest that generation of ROS determines bactericidal activity of LL-37 against *E. coli* and they demonstrated that intracellular accumulation of human cathelicidin influences the redox and ion status of bacterial cells^28^. It is assumed that similar phenomena occur in fungal cells treated with LL-37 and its non-peptide analogs. Validatoin of this hypothesis will require additional experimental work and analyses.

Apart from the achievements in the design of innovative fungicidal agents based on titanium dioxide, zinc oxide and silver nanoparticles, it was suggested that great potential lies within magnetic nanoparticles possessing theranostic potential. Iron oxide-based magnetic nanoparticles offer the possibility of development of multifunctional nanosystems aimed not only at eradication of pathogenic fungi, but also useful in the diagnosis of fungal infection or engaged as sensitizing tools in modern photodynamic therapy and potential magnetic fluid hyperthermia (MFH)-based treatments. It has been suggested that due to superparamagnetic properties of iron oxide-nanoparticles it is possible to employ these nanoparticles in magnetic fluid hyperthermia directed to eradication of fungal biofilms. So far, MFH-focused therapy has been engaged successfully in the therapy of bacterial biofilms, which involved the dispersal of pre-established biofilms at elevated temperatures^29, 30^. Moreover, there are a number of data demonstrating that MNPs conjugated with photosensitizers might be efficient in anti-biofilm photodynamic therapy. To date, the effectiveness of this therapy was confirmed mainly for gold nanoparticles; however, the reports demonstrating multifunctional nanosystems containing both iron oxide and gold nanoparticles conjugated with photosensitizers clearly indicate that the potential of MNPs in this therapy is significant^31^. In the case of fungal infections, which are a heterogeneous group of diseases and characterized by different courses of infection, the use of personalized therapy combined with real-time diagnostics and treatment progress, which is a central theme in nanomedicine. Given the above considerations, these studies focused on the employment of iron oxide-based nanoagents are justified and needed, despite other achievements in the development of novel fungicidal agents based on other types of nanoparticles.

Our previous research demonstrated the high activity of cationic lipids, especially synthetic steroid analogs, against laboratory *C. albicans* strains, clinical and environmental isolates of yeast or filamentous fungi such as *Aspergillus fumigatus*, *Blastomyces dermatitidis* and *Cryptococcus neoformans* ^23^. Currently we show that the same agents might be successfully used in the treatment of fungal infections caused by clinical strains of *C. albicans, C. glabrata, C. tropicalis* and other representatives of *Candida* spp. Importantly, their killing properties might be significantly enhanced by the functionalization of LL-37 peptide and CSA-13 on the surface of MNPs. It is likely that enhancement of the antimicrobial activity of LL-37 and CSA-13 by MNPs against fungal cells is caused by the ability of MNP-based nanosystem to penetrate cell membranes and increasing cellular uptake of antimicrobial agents, which is in agreement with previous reports indicating that MNPs can be employed as effective drug carriers for antimicrobial agents^13^. It is also possible that increased biological activty of such nanosystems is determined by enhanced ROS generation in treated fungal cells, which is due to the ability of magnetic nanoparticles to generat ROS. In our previous report, we demonstrated that aminosaline magnetic nanoparticles are able to penetrate *Candida albicans* cells, which was confirmed by TEM-based detection of intracellular vacuoles and endosome structures containing MNP@NH_2_ in treated fungal cells^21^. Moreover, our recent study performed using magnetic nanoparticles as drug delivery systems demonstrated enhancement of fungicidal activities of polyene antibiotics when incorporated into MNP-based nanosystems^26^. Fluorimetric-based analysis provided evidence that the level of ROS is considerably higher for fungal samples treated with CAPs-decorated nanosystems than their non-magnetic counterparts (data not shown). Importantly, the candidacidal activity of synthesized nanosystems is not affected by the presence of body fluids. Furthermore, the steady and controlled release of bioactive molecules incorporated into nanosystems sustains their antimicrobial activity compared to the free drug form^32^. Recent results show that CAPs decorated nanosystems possess the ability to control the release of ceragenin from the nanoparticle surface depending on environmental pH^22^. This property provides the opportunity to modulate CAP-decorated nanosystem activity in body fluids. Previous studies show that the biological activity of antimicrobial peptides, including cathelicidin LL-37, is considerably influenced by factors present in human body fluids including proteases, ions, pH, mucins, DNA, F-actin, apolipoprotein A and glycosaminoglycans^33–36^. Importantly, ceragenin CSA-13, due to its non-peptide character, maintains its biological activity in saliva, ascites, cerebrospinal fluid, bronchoalveolar lavage, pus, cystic fibrosis sputum, urine and is only partially inhibited by compounds present in serum^33, 37–40^. Immobilization of LL-37 peptide and CSA-13 on the aminosilane surface increases the killing activity of these compounds despite the presence of inhibitory factors. This is particularly advantageous in the case of LL-37, which is characterized by high MIC/MBC/MBIC values when used in the non-modified form, in contrast to its MNP-derivatives.

Modern antibiotic therapy is focused not only on killing the planktonic form of microorganisms, but also on the eradication of pathogens embedded into biofilms. Numerous factors are involved in the process of biofilm matrix formation including extracellular DNA (eDNA), polysaccharides and/or host F-actin. Biofilm formation is an emerging problem that impedes effective antimicrobial therapy, facilitates chronic infection and promotes drug resistant fungal strains^6, 41^. Nagant *et al*. demonstrated that ceragenin CSA-13, due to its membrane permeabilizing properties, acts as killing agent on established biofilms formed by *Pseudomonas aeruginosa* ^42^. Previous research by Moscoso *et al*. shows that ceragenins might also be used for the treatment of pathogenic streptococci, which is in agreement with other studies reporting high anti-biofilm activity of this compound^39, 43, 44^. Our study shows that LL-37 peptide and CSA-13 strongly inhibit the adhesion and formation of fungal biofilms, a property that may be enhanced through immobilization of these agents on MNPs. The anti-adhesive properties of the presented nanosystems suggest a role for coating in the prevention of fungal infection associated with medical device colonization. Interestingly, unmodified nanoparticles are known to strongly inhibit *C. albicans* biofilm formation due to cell wall disruption, resulting in the loss of fungal biofilm structure^45^. One may therefore assume that the enhanced anti-biofilm activity of magnetic LL-37/CSA-13 derivatives is due to a synergistic effect between iron-based nanostructures and cationic lipids with membrane permeabilizing properties. The proposed mechanism of synergistic action has been confirmed for cationic lipids when combined with different core-shell nanostructures with Gram-positive and Gram-negative bacteria^46^.

The attachment of CSA-13 to the MNPs significantly improves the biocompatibility of this compound, which is reflected by the lower level of LDH release from osteoblasts. Recent data indicate that nanoparticles with a magnetite core have increased blood retention time, low toxicity and biodegradability^47^, which suggests they may be effective carriers for antimicrobial agents. A report by Ruden *et al*. recently demonstrated a synergistic interaction between nanomaterials and membrane-permeabilizing agents without causing host cell damage^48^. Additionally, maintained viability of Hep-G2 cells following treatment with metallic nanoparticles in doses toxic to the tested microorganisms, including *Staphylococcus aureus* and *Pseudomonas aeruginosa* further confirms the high biocompatibility of nanostructures and reaffirms their potential use as drug delivery carriers^49^. Iron oxide-based nanosystems containing polyene antibiotics are characterized by better biocompatibility compared to free nystatin and amphotericin B^26^. The data presented in this study are in agreement with our previous research demonstrating decreased red blood cell hemolysis after treatment with MNP@CSA-13 compared to CSA-13 alone^22^. Importantly, both MNP@LL-37 and MNP@CSA-13 do not affect significantly proliferation of hFOB 1.19 cells even at high doses (250 µg/mL). In fact, the increased osteoblasts cell proliferation suggests that cationic lipids and their magnetic derivatives should be studied in the context of regenerative medicine. On the other hand, effects on tumor development and cancerogenesis should be investigated to assure safety. It was previously hypothesized that the alternations in membrane structure mediated by CAPs are due to cooperative binding to specific receptors and causes pleiotropic activity of these compounds with regard to the stimulation of cytokine release^50, 51^. However, the IL-8 release measurements confirmed that functionalization of cationic lipids on the surface of MNPs improves killing parameters of these agents, without significant effect on its immunomodulatory properties.

In conclusion, the data shown here establish the enhanced fungicidal activity of cationic lipids, particularly ceragenin CSA-13 and its magnetic derivatives, and point toward the development of novel antifungal agents with membrane permeabilizing properties. The ability to control the release of CAPs from nanosystems and improve efficacy might minimize the length of treatment and increase biocompatibility and lessening potential side effects. A shorter treatment duration and limitation of adverse effect would ultimately enhance patients’ compliance for treatment of fungal infection. Additionally our study indicates the increased potential of biocompatible nanosystems containing cationic lipids in the treatment of fungal infections caused by clinical *Candida* strains without affecting the immunomodulatory properties of these compounds.

## Methods

### Nanoparticle synthesis and characterization

To obtain aminosilane coated magnetic nanoparticles, co-precipitation of iron salts and then polycondensation of (3-aminopropyl)trimethoxysilane (APTMS) was carried out^52, 53^. In the next step, reaction with glutaraldehyde was performed to obtain a platform for CSA-13 immobilization via imine bonding^22^. In turn, functionalization of aminosilane modified magnetic nanoparticles by cathelicidin LL-37 was achieved by an amidation reaction between the peptide carboxyl group and the primary propyloamine group presented on the MNPs surface^54^. MNPs functionalized with LL-37 and CSA-13 were characterized by FT-IR spectroscopy using a Thermo Scientific Nicolet 6700 FT-IR spectrophotometer. The thermal properties including differential scanning calorimetry (DSC) and thermogravimetric analysis (TGA) were determined using a TA instruments USA (DSC Discovery; TGA Q500 Thermogravimetric Analyzer). Transmission electron microscopy TEM/EDX (Tecnai G2 X-TWIN) system was used to evaluate functionalized MNP size and morphology. The zeta potential was determined using the Zetasizer Nano ZS (Malvern Instruments, UK).

### Antifungal activity - evaluation of MIC, MFC and MBIC

To evaluate the minimum inhibitory concentration (MIC) of cationic lipids and their magnetic derivatives, the microdilution method was used in accordance with the guidelines of the Clinical Laboratory Standards Institute (CLSI). The final concentrations of the tested compounds ranged from 256 to 0.5 µg/mL. The MIC values were determined visually as the lowest concentration of tested agents that showed no microbe growth after 24–48 h of incubation. *In vitro* fungicidal (MFCs – minimum fungicidal concentration) activity was determined by plating each sample (10 µL) on Sabouraud dextrose agar plates. Minimum biofilm inhibitory concentration (MBIC) was assessed using an MTT test based on the reduction of tetrazolium salts. To determine the ability of the tested agents to kill fungal cells in different physiological conditions, MIC/MFC/MBIC measurements were performed in the presence of 50% human blood plasma, saliva, urine, pus, cerebrospinal fluid, abdominal fluid and dental plaque.

### Inhibition of fungal biofilm formation

*Candida spp*. biofilm was grown for 48 h at 37 °C in the presence of the tested agents. Three different clinical isolates of *Candida* spp. were used. Cathelicidin LL-37, ceragenin CSA-13 and their magnetic derivatives (MNP, MNP@LL-37 and MNP@CSA-13) was added at a concentration range of 1–100 μg/mL. Each well was washed 3 times with deionized water to remove planktonic cells. Biofilm mass was evaluated using 0.1% crystal violet (CV) staining. The stain was rinsed off with deionized water and plates were dried. Finally, 100 μL ethanol was added and the optical density (OD) was measured at a wavelength of 570 nm.

### Outer membrane permeabilization assay

N-phenyl-1-napthylamine (NPN) uptake assay was used to assess the outer membrane permeability of fungal cells. Fungal cells were resuspended in PBS (OD_600_ = 0.1) prior to incubation with cationic lipids (LL-37 and CSA-13) and their magnetic derivatives (MNP, MNP@LL-37 and MNP@CSA-13) in concentrations ranging from 1 to 20 µg/mL. NPN was added to a final concentration of 0.5 mM, and the mixture was incubated for 5 min. The increase in fluorescence intensity was recorded (excitation/emission wavelengths of 348/408 nm) using the Varioscan Lux microplate reader (Thermo Scientific).

### Measurement of fungal membrane affinity

To assess the affinity of cathelicidin LL-37, ceragenin CSA-13 and their magnetic derivatives (MNP, MNP@LL-37, MNP@CSA-13) towards fungal membrane, the tested compounds were labeled with fluorescein isothiocyanate (FITC) and added to cellular suspensions (OD_600_ = 0.1) to a concentration range 1–100 µg/mL. The fluorescence of the labeled compounds increases in a lipophilic environment during membrane permeabilization. Transfer of the FITC-labeled agents from the buffer to the pathogen membrane causes an increase in the light emitted at 534 nm following excitation at 298 nm. The affinity of labeled compounds to fungal cell membranes was assessed by fluorimetric measurement (Varioscan LUX Thermo Scientific) with excitation/emission wavelengths of 298/534 nm recorded for 10 min. Additionally, to show the ability of the nanosystems to penetrate into fungal cell, real-time confocal microscopic analysis with images recorded at 400× magnification was performed. Control cells were indicated by red color, and green/yellow color indicates cells after internalization of FITC-functionalized nanosystems.

### ROS generation assessment

Generation of ROS in *Candida albicans* cells was measured using 2′,7′-dichlorofluorescin diacetate (DFCH-DA) as a fluorescent probe. For this purpose, *Candida albicans* cells (OD_600_ = 0.7) were pipetted in to 96-well black plates with clear bottoms and then nanoparticles (MNPs, MNP@LL-37 and MNP@CSA-13) at concentrations of 10, 50 and 100 µg/mL were added to each well. DFCH-DA in PBS at 20 µM was then added. Fluorescence was measured for 60 min immediately after addition of the dye at excitation/emission wavelengths of 488/535 nm.

### Measure of the zeta potential of Candida albicans cells

The zeta potential measurements of *C. albicans* were taken at 25 °C on a Zetasizer Nano ZS (Malvern Instruments, UK) apparatus before and after treatment with 100 μg/mL of CAPs decorated magnetic nanoparticles. Details of the procedure were described previously^26^.

### AFM imagining

AFM images were taken using an AFM BioScope Catalyst (LABSOFT, Bruker Nano Surfaces Division, Santa Barbara, USA) working in contact mode. All measurements were performed in air-dried samples in accordance with previously described procedures^33^.

### Cell culture

Human osteoblast cells hFOB 1.19 (ATCC® CRL11372, ATCC, Manassas, Virginia, USA) were maintained in a 1:1 mixture of Ham’s F12 Medium Dulbecco’s Modified Eagle’s Medium, with 2.5 mM L-glutamine, without phenol red, supplemented with 10% fetal bovine serum (FBS), 50 U/mL penicillin, 50 mg/mL streptomycin and 0.3 mg/mL G418 in a humidified incubator containing 5% CO_2_ in air at 37 °C.

### Evaluation of IL-8 concentration and cytotoxicity using the hFOB 1.19 cell line

To evaluate interleukin-8 (IL-8) release and cytotoxicity after incubation of cells with cathelicidin LL-37, ceragenin CSA-13 and synthesized nanoparticles MNP@LL-37 and MNP@CSA-13, cells were seeded at a density of 5 × 10^4^ cells per well in 1 mL of growth medium in 24 well plates. After 24 h of culture, the cells were treated with the tested agents at a concentration range of 1–250 μg/mL and IL-8 and LDH assays were performed. IL-8 concentration was determined using an IL-8 ELISA kit (Invitrogen). The experimental averages are shown as percent cell viability compared to control, while cells treated with lytic solution were considered as 100% LDH release.

### Proliferation assay

To examine the effect of cathelicidin LL-37, ceragenin CSA-13, synthesized nanoparticles MNP@LL-37 and MNP@CSA-13 on hFOB 1.19 proliferation, cells were seeded in 24 well plates at 1 × 10^4^ cells per well with 1 mL of growth medium. Then the tested compounds were added to subconfluent cells at concentration 5–250 µg/mL. Cells were incubated for 24 h at 34 °C and then resazurin was added at a final concentration of 5 µg/mL. Fluorescence was recorded for 6 h with excitation/emission wavelengths of 520/590 nm.

### Ethics Statement

To perform evaluation of tested agents in the presence of the body fluids the materials were collected from adult healthy volunteers under IRB approval: R-I-002/575/2013 and R-I-002/382/2012. All methods were performed in accordance with the relevant guidelines and regulations and were approved by the institutional review board (IRB) of The Medical University of Bialystok. All subjects provided informed written consent and collected samples were anonymous.
